# The evolutionary biology of endometriosis

**DOI:** 10.1093/emph/eoab008

**Published:** 2021-03-12

**Authors:** Natalie Dinsdale, Pablo Nepomnaschy, Bernard Crespi

**Affiliations:** 1 Department of Biological Sciences, Simon Fraser University, Burnaby, BC, Canada; 2 Faculty of Health Sciences, Simon Fraser University, Burnaby, BC, Canada

**Keywords:** endometriosis, polycystic ovary syndrome, prenatal testosterone, diametric disorders, sexual dimorphis

## Abstract

We provide the first analysis and synthesis of the evolutionary and mechanistic bases for risk of endometriosis in humans, structured around Niko Tinbergen's four questions about phenotypes: phylogenetic history, development, mechanism and adaptive significance. Endometriosis, which is characterized by the proliferation of endometrial tissue outside of the uterus, has its phylogenetic roots in the evolution of three causally linked traits: (1) highly invasive placentation, (2) spontaneous rather than implantation-driven endometrial decidualization and (3) frequent extensive estrogen-driven endometrial proliferation and inflammation, followed by heavy menstrual bleeding. Endometriosis is potentiated by these traits and appears to be driven, proximately, by relatively low levels of prenatal and postnatal testosterone. Testosterone affects the developing hypothalamic–pituitary–ovarian (HPO) axis, and at low levels, it can result in an altered trajectory of reproductive and physiological phenotypes that in extreme cases can mediate the symptoms of endometriosis. Polycystic ovary syndrome, by contrast, is known from previous work to be caused primarily by high prenatal and postnatal testosterone, and it demonstrates a set of phenotypes opposite to those found in endometriosis. The hypothesis that endometriosis risk is driven by low prenatal testosterone, and involves extreme expression of some reproductive phenotypes, is supported by a suite of evidence from genetics, development, endocrinology, morphology and life history. The hypothesis also provides insights into why these two diametric, fitness-reducing disorders are maintained at such high frequencies in human populations. Finally, the hypotheses described and evaluated here lead to numerous testable predictions and have direct implications for the treatment and study of endometriosis.

**Lay summary:** Endometriosis is caused by endometrial tissue outside of the uterus. We explain why and how humans are vulnerable to this disease, and new perspectives on understanding and treating it. Endometriosis shows evidence of being caused in part by relatively low testosterone during fetal development, that ‘programs’ female reproductive development. By contrast, polycystic ovary syndrome is associated with relatively high testosterone in prenatal development. These two disorders can thus be seen as ‘opposite’ to one another in their major causes and correlates. Important new insights regarding diagnosis, study and treatment of endometriosis follow from these considerations.

## INTRODUCTION

Human diseases and disorders have ultimate, evolutionary causes as well as proximate, mechanistic ones. Evolutionary causes of disease include evolutionary legacies (which can constrain adaptation and potentiate maladaptation), tradeoffs (which can prevent optimization of structure or function), mismatches to modernity (which can cause maladaptation via divergence between phenotypes and environments), genetic conflicts (which can cause or generate new scope for maladaptation) and benefits to reproduction that are coupled with health-related costs to the self [1]. Integrated analyses of the proximate and ultimate causes of disease can deepen insights into both disease etiology and evolutionary processes. Such studies are especially important when diseases are human-specific or human-elaborated, and derive, in part, from effects of recent selection along the lineage leading to modern humans.

In this article, we synthesize ultimate with proximate causes in the study of endometriosis. We first describe the symptoms and diagnostic features of endometriosis, and review previous hypotheses on its physiological determinants. Second, we analyze the bases for endometriosis by applying Niko Tinbergen's [2] four questions for analyzing phenotypes: (1) phylogeny and evolutionary history, (2) development, (3) mechanism and (4) adaptive significance. Addressing these four questions allows for an integrative, interdisciplinary analysis of the causes and correlates of endometriosis and the traits that characterize it, in the context of understanding why and how this disorder persists in humans given its high heritability. In doing so, we also describe, evaluate and provide tests of a recently developed hypothesis for a primary cause of endometriosis: that its development is driven by relatively low levels of testosterone in prenatal development [3].

## ENDOMETRIOSIS

Endometriosis is found almost exclusively in women. For the purposes of this article, we use the terms ‘women’ and ‘men’, and ‘females’ and ‘males’ (in the context of humans), to refer to the sex of individuals of *Homo sapiens* who bear XX and XY sex chromosomes respectively, with a functional SRY gene in males. Other chromosome complements exist in humans (such as XXY, and XX/XY mosaicism) but are not considered here. These definitions hold regardless of an individual's gender, which involves aspects of culture.

Endometriosis, which is found in 5–10% of reproductive-aged women, is characterized by endometrial tissue becoming established and growing outside of the uterus, most commonly in the ovaries, pelvic cavity, fallopian tubes, or rectovaginal area [4] (Boxes 1 and 2). Such ‘ectopic’ tissue undergoes menstrual cycle changes like normally situated, ‘eutopic’ endometrium, proliferating under the influence of estradiol, and breaking down during menstrual periods, but with no means of exiting the body. Endometriosis can cause severe pelvic pain, as well as contributing to reduced fertility in a substantial proportion of patients. There is no cure, and management typically involves damping or shutting down the estrogenic stimuli that promote ectopic endometrial tissue proliferation (as well as normal cycling and fertility), surgery to remove the lesions or hysterectomy.

Two main hypotheses have been proposed to help explain the proximate basis of human vulnerability to endometriosis. First, as described by Sampson [10] who originally described and named the disease, endometriosis may be caused by ‘retrograde flow’: endometrial cells that become misplaced during menstruation and establish ectopically. This hypothesis can provide only a partial explanation for endometriosis because at least 90% of women undergo some degree of retrograde menstruation [11], but a much smaller percentage develop the disease [12]; moreover, some endometriotic sites are not accessible to cells via retrograde menstruation. Second, ectopic endometrial tissue may derive from female reproductive system (Müllerian duct) cells that undergo altered differentiation, migration or both, during early *in utero* development [13]. This hypothesis can explain ectopic lesions at diverse bodily sites other than the peritoneal cavity, as well as (extremely rarely) in males (given that males have Müllerian ducts in early development), and it is consistent with results from some histological studies [13]. However, its general validity remains unknown. The proximate causes of endometriosis thus remain enigmatic, and its ultimate, evolutionary causes have not been addressed previously in any detail.

We propose here that Tinbergen's four questions, taken together in the context of core evolutionary-medical principles, can provide novel and useful insights into the causes and treatments of endometriosis. We consider each question in turn.

## PHYLOGENY AND EVOLUTIONARY HISTORY

Tinbergen's first question focuses on how a trait evolved. Evolutionary histories are important to understanding disease because they generate the potential and scope for maladaptations that can manifest as disease and its symptoms. In this context, humans are unusual among mammals in that they exhibit a prominent menstruation process including the monthly discharge of blood and other components of degraded endometrial tissue. The presence of menstruation is rare in mammals, being restricted to some primates, a few bats, elephant shrews and one rodent (the spiny mouse) [14]. Menstruation has been reported only among mammals that exhibit highly invasive (hemochorial) placentation [15], and the comparative association of menstruation with invasive placentation has been interpreted in the context of maternal–fetal conflicts [16]. By this hypothesis, the placenta (which is derived from the fetus) has in some lineages, and especially in humans, evolved relatively deep invasion of endometrial tissue, and spiral artery modification, to enhance resource acquisition by the fetus [17]. Mothers have concomitantly, in response, evolved a more highly developed, thicker endometrium, that decidualizes (differentiates, under the influence of progesterone) spontaneously in preparation for implantation, instead of being induced by implantation, as in most mammals [15, 16, 18]. If implantation does not occur in any given menstrual cycle, the endometrial tissue breaks down.

The unusual uterine endometrial buildup cycle and menstrual bleeding pattern humans may generate risk for endometriosis in several ways:

1. shed endometrial cells can undergo retrograde flow, as described above, involving relatively high volumes in humans, thus increasing the risk for establishment of ectopic endometrial tissue [11, 12];
2. endometrial tissue undergoes especially rapid and prolonged proliferation every month during the follicular phase of cycling in humans, such that specialized physiological pathways support its estrogen-fueled growth [19], in both eutopic and ectopic tissues;
3. breakdown of the endometrial tissue with menstruation is a highly inflammatory process [20], generating the potential for widespread inflammation of endometrial cells at ectopic sites (as seen in endometriosis) that can be facilitated by high estradiol and low testosterone (e.g., [21]) and be unopposed by anti-inflammatory signals [22]; and
4. embryo implantation into endometrial tissue is also inflammatory, because the fetal-placental unit penetrates deeply into the decidualized endometrium for successful establishment of pregnancy. These observations raise the possibility that in endometriosis, endometrial cells may implant ectopically in ways that facilitate inflammation [23], which is a normal part of their interaction with other tissues including the fetal-placental unit. For example, implantation of embryos in eutopic endometrial tissue can be facilitated by ‘scratching’ (local surgical injury), which elicits inflammation [24]; in parallel, ectopic endometrial tissue (endometriosis) preferentially implants and develops at similarly impacted sites, such as scars from recent surgery [23].

Among nonhuman animals, endometriosis has been reported only among primates that menstruate [25]. Other great apes exhibit invasive placentation as well as menstruation, and endometriosis, or the closely related disease adenomyosis, has been reported in orangutans, chimpanzees and gorillas [26]. However, the prevalence of these disorders in these and other nonhuman primates remains unknown, mainly because diagnosis requires the invasive procedure of laparoscopy (or autopsy).

Several other human-evolved traits may also increase endometriosis risk. First, in humans, the endometrial proliferative phase of the menstrual cycle is especially long, placing the later stages of follicle selection (from many small follicles to a single dominant one) entirely within the follicular stage. This heterochronic change in menstrual cycle timing, along the human lineage, generates the potential for relatively high levels of luteinizing hormone (LH), in some individuals, to generate excessive levels of ovarian androgens that can stall follicle development in the polyfollicular stage and prevent ovulation [15]. Relatively low LH and low ovarian androgen levels may also impact follicular development, though in different ways (e.g., [27]), as described in more detail below. Second, selection along the human lineage for more-intense uterine contractions during childbirth, due to large fetal head circumference, may have pleiotropically led to risk of stronger contractions during menstruation, which is represented as dysmenorrhea in endometriosis [28].

In sum, the human menstrual cycling is characterized by an unusual set of traits. Understanding the molecular mechanisms of rapid endometrial growth, inflammatory signaling in menstruation and implantation, anti-inflammatory signaling after successful implantation, spontaneous, compared to induced, decidualization and uterine contractility during menstruation and childbirth should provide important information concerning risk and causes of endometriosis. These considerations also suggest that current experimental systems for the study of endometriosis (e.g., laboratory mice with endometrial cells inserted ectopically) make limited or misleading models due to their divergence from humans in key aspects of reproductive physiology. The only menstruating rodent, spiny mice, provides an alternative that may be more similar reproductively to humans and may thus provide a better animal model of endometriosis [14].

## ONTOGENY

Tinbergen's second question refers to phenotypic development. Here, the phenotype is, in a broad sense, represented by women's reproductive morphology and physiology, which begins to develop *in utero* during the first 12 weeks of gestation. This period is characterized first by the divergence between sexes, and next, for females, by development of the HPO axis, which is orchestrated by neurological, hormonal, homeobox-based systems and other transcription factor signaling programs, that determine how it will operate during later life [29]. Early sexual developmental effects, especially early effects of prenatal testosterone, are important because they determine how and why the female HPO axis may become subject to early dysregulation that increases risks for endometriosis and other reproductive disorders.

Sex differentiation is mediated by expression of the transcription factor SRY, that upregulates Sox9, and leads to testosterone production by Leydig cells of the testis, regression of the Müllerian ducts, and development of the testis and other external male genitalia. By contrast, in females, the Wolffian ducts regress and the Müllerian ducts, plus the urogenital sinus, develop into the external genitalia, in part due to antagonism of Sox9 by Foxl2, Wnt4 and Dax1. Differentiation and differences in phenotypes between females and males, with regard to gonads, other bodily systems, the brain and genetic complements, are not dichotomous and deterministic, but show variation along continua that is mediated by a wide range of biological and cultural factors; in this context, each sex also varies in these traits to a considerable degree.

Testosterone is present and active prenatally among females (at lower levels than among males), being produced from fetal and maternal adrenal glands, and maternal ovaries and maternal fat tissue, with maternal sources reaching the fetus via the placenta [30]. Testosterone levels also vary prenatally among females, as evidenced, for example, by effects on sexually dimorphic facial morphology in humans [31], and by extensive experimental studies of other mammals including cows, sheep, mice, monkeys, rats and rabbits (e.g., [32, 33]).

Among both females and males, prenatal testosterone levels have been linked with two main anatomical metrics. Anogenital distance (AGD), usually measured from the scrotal base to the anus in males, or from the distal end of the vagina to the anus in females, is much longer on average in males than females. Longer AGD is strongly associated with higher prenatal testosterone in both sexes [34], with considerable variation within each sex. A second metric of prenatal testosterone, the ratio of the 2nd to the 4th finger length (2D4D digit ratio) is lower among males than females, and lower digit ratio indexes higher prenatal testosterone within each sex as well, though with considerable variability [35]. Some females thus develop under conditions of lower, or higher, prenatal testosterone than others, and lower prenatal testosterone involves a shorter AGD, longer 2D4D and notable effects on development of the HPO axis.

The relevance of within-sex prenatal testosterone variation effects to development of the female HPO axis is clearly indicated by well replicated associations of relatively high prenatal testosterone with the development of polycystic ovary syndrome (PCOS) [36, 37]. PCOS is characterized by anovulation, polyfollicular (polycystic) ovaries, and high serum and ovarian testosterone (Box 2). These traits are associated with the development of phenotypes including hirsutism (increased hair growth on the face, chest and back) and a predominance of abdominal rather than gluteofemoral distribution of fat, which leads to high body mass index (BMI) and waist to hip ratio (WHR) [38]. The HPO axis is altered in PCOS in that GnRH (gonadotropin-releasing hormone) pulsatile secretion rates are increased, leading to higher LH relative to FSH (follicle stimulating hormone), higher AMH (antimüllerian hormone) and lower SHBG (serum hormone binding globulin) in conjunction with higher postnatal serum testosterone and lower oxytocin [3].

PCOS is thus a developmental disorder of the HPO axis, that is mediated by relatively high prenatal testosterone and other factors [36, 37]. AGD is higher in adult women with PCOS compared to controls [3, 39] and in the fetuses of women with PCOS (as measured with ultrasound), and 2D4D digits ratios are lower in women with PCOS [3], all of which demonstrate the importance of higher prenatal testosterone in the etiology of this condition.

Endometriosis, in contrast to PCOS, exhibits a diverse set of hormonal indicators, correlates and risk factors suggesting that it is ‘opposite’ to PCOS in its causes and symptoms [3]. Most notably, women with endometriosis exhibit, relative to controls: (1) shorter AGDs, indicative of lower prenatal testosterone [40, 41], (2) lower LH relative to FSH, (3) higher SHBG, (4) higher serum oxytocin, (5) lower ovarian and serum testosterone, (6) lower AMH, (7) lower WHR and BMI, (8) lower β-endorphin and (9) a suite of other differences, all of which are opposite to the differences found between PCOS versus controls [3]. The patterns of differences between women with PCOS and endometriosis are highly concordant with one another: for example, in women without PCOS or endometriosis, higher postnatal serum testosterone has been associated with lower SHBG, longer AGD, higher LH, lower FSH, higher β-endorphin and higher WHR (e.g., [3, 42, 43]).

**Figure 1.**
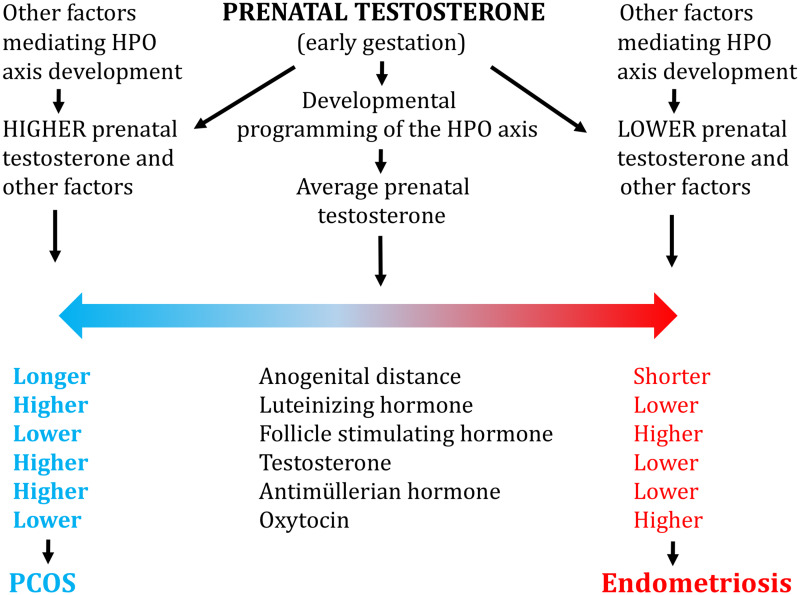
Model for how prenatal testosterone mediates risks of endometriosis and PCOS, through effects on major hormones that regulate the female HPO axis. Endometriosis and PCOS thus represent extremes of a continuum of HPO programming effects driven by low versus high prenatal testosterone, respectively. See text for details.

Endometriosis and PCOS thus appear to represent an excellent example of diametric (opposite) disorders such as osteoporosis versus osteoarthritis, pre-eclampsia versus post-partum hemorrhage, and cancer versus neurodegeneration, that are mediated by the tendency for biological systems to vary in two directions in expression or activation, with disorders presenting at the two extremes (e.g., [3, 44, 45]). Like PCOS, the diagnostic features of endometriosis are aligned along a graded continuum, from high severity, to mild forms, to the presence of some endometriosis-associated traits, and to an absence of the disease. By the diametric model, then, endometriosis and PCOS represent the two polar extremes and endpoints of a continuum of reproductive physiological traits that are related, in part, to prenatal testosterone (Fig. 1).

Taken together, the findings described above for endometriosis and PCOS provide evidence that the ontogeny of endometriosis is linked with the quantitative expression of prenatal factors, especially testosterone (and possibly testosterone relative to estrogen), that influence aspects of human sexual dimorphism [3]. As such, the causes, effects and correlates of endometriosis may also be characterized by alterations, and extreme values, in quantitative traits that differ between males and females, and among females, as shown for example by the menstrual, immune, endocrine and physical phenotypes in Table 1.

**Table 1.** Evidence salient to the hypothesis that endometriosis involves relative extremes of phenotypes that show evidence of sex differences in humans, due to genetic and environmental effects during development

Trait	Female–male sex difference, or variation among females	Pattern in endometriosis or its symptoms	References
Anogenital distance	Shorter in females than in males	Shorter in women with endometriosis compared to control women	[40, 41]
2D4D digit ratio	Longer in females than in males	Longer in women with heavy menstrual bleeding and dysmenorrhea (menstrual pain during periods caused by contractions)	[46]
Waist-to-hip ratio	Lower in women than in men	Lower in women with endometriosis than in control women	[47]
Inflammation levels	Higher in women than in men, during reproductive period of female life history	Higher in women with endometriosis compared to control women and men	[21, 48]
Pain (chronic)	Higher sensitivity in women than in men, and lower β-endorphin levels in women	Higher sensitivity to pain, and lower β-endorphin levels, in women with endometriosis compared to controls	See text for details
Serum testosterone	Lower in women than men	Lower in women with endometriosis than in controls	[49]
Serum oxytocin	Higher in women than in men	Higher in women with endometriosis than in control women	[50, 51]
Timing of menarche and menopause	Varies among females	Earlier menarche and menopause in women with endometriosis	[52, 53]
Length of menstrual cycle	Varies among females	Shorter menstrual cycle length in women with endometriosis	[54]
Dysmenorrhea	Varies among females	Higher levels in women with endometriosis	[55]
Bleeding during menstruation	Varies among females	Higher levels in women with endometriosis	[55]

Pain, which represents one of the primary symptoms of endometriosis, represents a noteworthy example of the interfaces between human sexual dimorphism, hormones and endometriosis. Women report higher sensitivity to pain on average than do men [56, 57], as well as showing lower serum levels of β-endorphin [58], and pain sensitivity is higher under lower levels of serum testosterone in both sexes [59]. In women, testosterone decreases pain perception, and estradiol increases it [60], and in rats, testosterone increases pain thresholds [61] and the androgen receptor antagonist flutamide decreases them [62]. Female rats treated prenatally with testosterone also show pain responses similar to those of untreated males, indicating that pain sensitivity is programmed, in part, during early development [63].

Women with PCOS exhibit higher serum testosterone, as well as higher β-endorphin levels and higher pain thresholds, compared to control women [64]. By contrast, women with endometriosis exhibit lower β-endorphin levels [65], and higher reported sensitivity to pain [66]. Moreover, treatment of endometriosis with the synthetic androgen danazol leads to substantial reductions in chronic pain symptoms [67]. These findings indicate that studying sex differences in pain, and their hormonal bases, should provide novel insights into its etiology and treatment in endometriosis.

The evolutionary developmental framework proposed here for explaining the phenotypes that characterize endometriosis is concordant with the two traditional hypotheses for its risk. Thus, increased levels of retrograde menstruation are expected from the more extreme expression of menstrual characteristics in endometriosis (especially increased menstrual flow), and displacement and altered differentiation of Müllerian stem cells may occur more easily under conditions of lower prenatal testosterone and a more pronounced expression of sexual differentiation during early prenatal development. One possible cause of such effects is the homeobox gene HOXA10, which orchestrates early development of the uterus, as well as endometrial functions, and whose expression is strongly affected by testosterone (e.g., [68]).

## MECHANISMS

Tinbergen's third question refers to the causation of a phenotype, which can be considered to encompass its genetic, environmental and physiological causes. We discuss each in turn.

### Genetic causes

Endometriosis risk is polygenic, with risk affected by many alleles each of small effect, and a heritability of around 0.5 [69]. Genome wide associations studies have identified a set of alleles (SNPs) that account for a small proportion of this heritability, and that includes genes associated with early sexual development (WNT4, HOXC6), steroid hormone signaling (ESR1, GREB1, KDR), overall growth (IGF1), the HPO axis (FSHB) and other traits [70].

Of the genome-wide significant endometriosis risk genes in the most recent GWAS [70], FSHB emerges as one of special interest because this gene regulates central aspects of female reproduction. The FSHB genetic risk factor for endometriosis involves a large (∼130kb) haplotype with two common alleles at frequencies of about 85 and 15% in European populations [71]. Compared to the minor (lower frequency) haplotype, the major FSHB haplotype has been associated across a large set of studies with higher FSH and lower LH (accounting for 3.5% and 7.1% of their variation respectively, in a typical population), higher risk of endometriosis, a large suite of endometriosis-associated and other traits, and lower risk of PCOS [72–79] (Table 2). This FSHB haplotype thus represents a remarkable example of a specific genetic factor with opposite effects on endometriosis and PCOS risks and their major features. At the whole genome level, endometriosis risk is also genetically correlated (associated via pleiotropy or linkage disequilibrium) with many of its major phenotypic correlates, including earlier menarche, earlier age at first birth, extreme menstrual bleeding, and earlier menopause [70, 81].

**Table 2.** Differences in trait expression between women with major versus minor haplotypes of FSHB promoter polymorphism marked by the SNP rs10835638 and tightly linked SNPs

**Type of trait**	**Pattern found in major haplotype compared to minor haplotype**	**Comments**
Endocrine	Lower luteinizing hormone Higher follicle-stimulating hormone Lower serum testosterone	All of these phenotypes are associated with endometriosis
Menstrual	Earlier menarche Shorter menstrual cycles More-excessive menstruation Earlier menopause	All of these phenotypes are associated with endometriosis
Reproductive	Earlier age at first parturition/birth Higher lifetime parity (births) Lower risk of nulliparity (no births) Higher rate of dizygotic twinning	Higher rate of dizygotic twinning may be due to higher rate of two luteinizing hormone surges (which typically indicate ovulation), in women with endometriosis [81]
Disease-related	Higher risk of endometriosis Lower risk of PCOS	Rates of endometriosis are also reduced among women diagnosed with PCOS, and involve minimal-to-mild expression (see [3])

See text for citations. In PheWAS analysis [80], rs10835638 is also associated with age at first live birth (*P* = 0.00031), WHR (*P* = 0.0016), number of older siblings (*P* = 0.0086), age at last live birth (*P* = 0.012), having had a bilateral oophorectomy (both ovaries removed) (*P* = 1.09 x 1 0 ^−10^) and having had a hysterectomy (removal of the uterus) (*P* = 5.6 x 1 0 ^−8^).

If low versus high prenatal testosterone contributes to the development of endometriosis versus PCOS [3], then genetic markers for correlates of prenatal testosterone (including AGD and 2D4D) should be associated with correlates of these two conditions. This hypothesis can be evaluated using results from the latest GWAS of a correlate of prenatal testosterone, 2D4D digit ratios, in humans, that reported twelve genome-wide significant SNP risk factors ([82]).

Four of the genes harboring digit ratio SNPs (EFNA1, HOXD12/HOXD11, GLI3 and SALL1) show evidence of altered expression in endometriosis; one digit ratio SNP is in the gene TOX3, which is genome-wide significant for risk of PCOS [83], two SNPs are in genes (SMOC1 and HOXA12) that regulate gonad development, and an interaction of HOXA12 with GLI3 mediates development of the vagina and uterus. PheWAS analysis [84] of the twelve SNPs associated with digit ratios, which shows evidence of pleiotropy in their effects, indicates that four of the 12 are associated at *P* < 0.05 with age at menarche, four with WHR, and nine with BMI (https://atlas.ctglab.nl/PheWAS), suggesting genetically based links of prenatal testosterone with each of these phenotypes, which fits well with the associations of these traits with endometriosis and PCOS at the level of phenotypes.

Concordance among the genetic and phenotypic correlates of endometriosis, in relation to PCOS, can also be analyzed by determining the degree to which disorder risk genes and SNPs are associated with disorder-related phenotypes. Four of 25 total, genome-wide associated endometriosis risk genes (Table 2 in [70]), and three (of 14) PCOS risk genes, are also associated with age of menarche or menopause; moreover, seven endometriosis risk genes, and five PCOS risk genes ([83]), were also associated with WHR or BMI (using Open Targets Genetics Total Association Scores, from https://www.targetvalidation.org). PheWAS analysis ([85], from https://genetics.opentargets.org/) of risk SNPs demonstrated comparable patterns: eight of 27 genome-wide significant endometriosis risk SNPs, and six genome-wide significant PCOS risk SNPs, were pleiotropically associated with age of menarche or menopause (or both, for FSHB and THADA SNPs), and five endometriosis risk SNPs, and two PCOS risk SNPs, were pleiotropically associated with WHR or BMI. Pleiotropic associations have also been reported for endometriosis with WHR [86] and with age at menarche [87]. Remarkably, PheWAS analysis also shows that for endometriosis, 11 (41%) of 27 genome-wide risk SNPs were associated with oophorectomy or hysterectomy. These results provide evidence that, as for effects of the FSHB haplotype described above, genetic risk factors for endometriosis and PCOS are closely associated with the life history and physical-feature correlates of these disorders.

### Environmental causes

PCOS is well known to be mediated by increased prenatal testosterone from environmental causes, such as experimental androgen administration in animals or gestational effects of having a mother with PCOS and high serum testosterone [36, 37]. By the hypothesis evaluated here, risk of endometriosis should be associated with lower testosterone, and with higher relative effects from estrogens, during prenatal development. This hypothesis is supported by links of endometriosis with a shorter AGD as described above, and it can be evaluated further using data from studies of prenatal exposures to antiandrogenic and estrogenic endocrine-disrupting chemicals in animals and humans, which are expected to increase risk of endometriosis and expression of its phenotypic correlates including shorter AGD and earlier menarche or estrus, and decreased risk of PCOS. Postnatal exposures may also affect endometriosis or PCOS risk, but are not necessarily relevant to the hypotheses addressed here because they do not involve prenatal programming of the female HPO.

Three endocrine-disrupting agents have been linked with prenatal effects on risk of endometriosis. First, prenatal exposure to the potent synthetic estrogen diethylstilbestrol (DES) is significantly associated with higher endometriosis risk in offspring [88].

Second, prenatal exposure to bisphenol A (BPA), an estrogenic chemical acquired in the diet, leads to shorter AGD in female rats [89], and is associated with earlier first estrus and the generation of endometriosis-like lesions in mice [90–92]. In humans, prenatal BPA leads to shorter AGD in female offspring, when exposure occurs during the first trimester [93].

Third, exposure prenatally to relatively high levels of phthalates, a family of mostly antiandrogenic agents, has been associated with shorter AGD in mice and humans [94–96]. Phthalate levels of women prior to 20 weeks of pregnancy have been linked with increased estradiol and decreased testosterone in infants [97]. Prenatal phthalate exposure has also been associated with reduced risk of PCOS in adolescent female offspring [98], which is consistent with their antiandrogenic effects. Endometriosis has been linked with higher levels of some phthalates in a meta-analysis [99], but none of the relevant studies involved prenatal exposures. Taken together, these studies of DES, BPA and phthalates strongly support the hypothesis that prenatal exposures to evolutionarily novel antiandrogenic and estrogenic chemicals increase risk of endometriosis.

### Physiological causes

The findings described above link endometriosis, and its correlates, with effects from reduced prenatal testosterone, due to both genetic and environmental factors. How do these effects translate, mechanistically, into the causes and correlates of endometriosis, in the context of the effects from higher prenatal testosterone that are established for PCOS?

In PCOS, hypothalamic sensitivity to steroid-induced negative feedback is reduced, resulting in an increased frequency and amplitude of GnRH and LH pulses with corresponding increases in levels of LH [100, 101]. Increased LH relative to FSH results in elevated androgen production and arrest of follicular maturation [102]. Immature follicles release high levels of AMH, which increase GnRH pulsatility and inhibit FSH [103]. The capacity to mount an LH surge is thereby impaired, so ovulation is interrupted (leading to anovulation or oligo-ovulation) and menstrual cycles lengthen or disappear [101].

In endometriosis, by contrast, lower LH relative to FSH is associated with lower AMH, signaling lower ovarian reserve and lower ovarian testosterone [3]. In the ovaries, a higher proportion of primordial follicles is recruited into the growing pool and a greater number of maturing follicles degenerate, resulting in quicker depletion of ovarian reserve [104]. Women with endometriosis have fewer pre-ovulatory follicles as well as smaller follicles, relative to unaffected women [105]. Low testosterone predicts elevated pro-apoptotic factors in the follicular fluid of women with endometriosis, which contributes to increased follicular atresia in affected women [49]. Some women with endometriosis also demonstrate premature follicle rupture, which contrasts to the stalled follicles that become cysts in PCOS [106].

**Figure 2.**
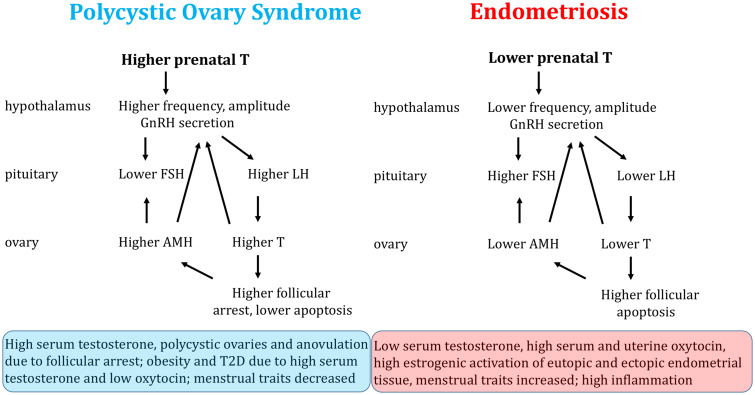
Models showing how risks for PCOS and endometriosis are mediated by effects of higher versus lower prenatal testosterone on development of the female HPO axis, and how these effects may generate the symptoms of each condition. Details of the mechanistic links of testosterone and other factors with ectopic endometrial implantation, proliferation and inflammation remain to be discerned.

Figure 2 provides a simple depiction of how HPO functions appear to differ between women with endometriosis compared to those with PCOS. Most of the patterns and causal connections shown have been demonstrated from previous work, with notable exceptions. These data gaps include: (1) a paucity of evidence regarding LH pulse frequency and amplitude in women with endometriosis, (2) incomplete knowledge regarding the mechanisms by which ovarian alterations in endometriosis, especially low testosterone and low AMH, influence endometrial function, particularly the establishment, growth and inflammation of endometrial tissue at ectopic sites, and (3) lack of clarity on how variation in ovarian or serum testosterone levels influences eutopic and ectopic endometrial tissues. For example, androgens have recently been shown to regulate the repair of endometrial tissue [107]; Could low levels in women with endometriosis contribute to both high inflammation and reduced repair of ectopic implants, that resemble ‘wounds that never heal’?

## ADAPTIVE SIGNIFICANCE

Tinbergen's fourth question refers to the adaptive significance of a phenotype, in terms of its effects on reproductive success, or fitness. Reproductive disorders like endometriosis or PCOS reduce fertility and fecundity as well as health, so application of this Tinbergen’s criterion also requires considering the fitness-related effects of variation in the nonclinical phenotypes or genotypes that are associated with these two conditions, in populations of women without either disorder. For endometriosis, these effects are known to involve a diverse set of findings:

1. The major FSHB haplotype associated with higher endometriosis risk is also connected to earlier age at first birth, higher lifetime parity, lower risk of nulliparity (no pregnancies) and a higher rate of dizygotic twinning, as noted above and in Table 2;
2. A Neanderthal-derived PROGINS progesterone receptor gene haplotype is associated with higher risk of endometriosis, and also with a lower rate of early miscarriage, and having more sisters [108, 109];
3. A higher 2D4D digit ratio, indicative of lower prenatal testosterone, has been associated with higher reproductive success of women in some populations [110, 111]; and
4. A lower WHR, as found in endometriosis, positively predicts conception rates [112] and has been associated with measures of higher fecundity and reproductive value in some studies [113–115];

Some animal studies are also relevant to the fitness-related effects of endometriosis-associated traits, even though the subject species do not develop endometriosis in the wild. Thus, in mice and rabbits, females that develop naturally under lower exposure to prenatal testosterone have shorter AGDs and higher fecundity, as well as being preferred by males for mating, compared to females that develop under higher testosterone levels [116–122]. These findings represent comparative evidence that low prenatal testosterone and shorter AGD, as also found in women with endometriosis, are associated with higher fecundity.

Considered together, these lines of evidence suggest that some endometriosis-associated genotypes and phenotypes are associated with increased fertility and fecundity. However, having too many such traits, or too high a level of their expression, apparently leads to maladaptive extremes, and to the lower reproduction observed in endometriosis itself.

How are PCOS-related genotypes and phenotypes associated with fertility and fecundity? The available evidence suggests that, unlike for the correlates of endometriosis, correlates of PCOS are associated with reduced fertility and fecundity; thus:

1. The minor FSHB haplotype is associated with PCOS risk and higher risk of nulliparity, as noted above;
2. Higher serum testosterone is associated with lower fertility, in a nonclinical population of women [123];
3. Women with male co-twins, and thus higher prenatal testosterone, exhibit evidence of lower fecundity than women with female co-twins [124, 125]; and
4. Higher WHR and BMI, as found in PCOS, are associated in some studies with lower indicators of fecundity and reproductive value (e.g., [115, 126–128]);

Animal studies also demonstrate that mouse and rabbit females developing naturally under exposure to higher prenatal testosterone have longer AGDs and lower fecundity, the opposite of the pattern described above for endometriosis. In addition, females with longer AGDs exhibit lower fertility or fecundity in pigs [129] and cows [130]. These studies of humans and nonhuman animals suggest that PCOS-related genotypes and phenotype are associated with lower fertility and fecundity, in contrast to the findings for endometriosis.

These considerations regarding adaptive significance bear directly on hypotheses for explaining the apparently paradoxical maintenance of endometriosis and PCOS in human populations, given that both of them are highly heritable, persist at frequencies of 5–10%, and substantially reduce reproduction. The maintenance of PCOS has been postulated to be the result of three possible factors. First, PCOS-related physiological traits, which can involve higher WHR and higher levels of visceral, ‘survival fat’, may have provided survivorship benefits in past environments with high fluctuations in food availability and periodic famines, but are deleterious in current environments with plentiful food [131–133]. Second, formerly neutral alleles may have become deleterious in the current, recent human obesogenic environment, generating PCOS phenotypes via maladaptive evolutionary mismatch [9, 134]. And third, PCOS may be maintained by sexually antagonistic selection, whereby selection favors higher fetal testosterone among developing males but disfavors it among developing females, with strong genetic or environmental correlations between the sexes preventing sex-specific optimization [135, 136].

The maintenance of endometriosis may be mediated in part by the fertility and fecundity effects described above, as well as by evolutionary mismatches. Two mismatch models have been proposed. First, risk may be increased by earlier menarche and later childbirth contributing to a higher number of menstrual cycles, that increase risk through more retrograde flow and more-frequent estrogenic stimulation of endometrial tissue [137, 138]. Second, prenatal exposure to anthropogenic estrogenic and antiandrogenic endocrine disrupting chemicals may substantially increase risk, as described above.

**Figure 3.**
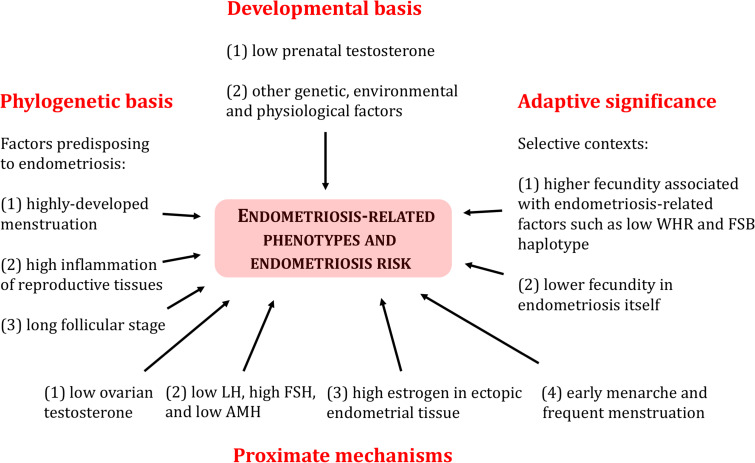
A summary of Tinbergen's four questions as regards understanding evolved risk, development, adaptive significance and mechanisms of endometriosis and its associated phenotypes

Figure 3 summarizes current understanding of Tinbergen's four questions, as regards endometriosis and the specific phenotypes that characterize it. Insights gained from this interdisciplinary approach provide a robust framework for study of the evolutionary medicine of endometriosis, using disciplines that range from genetics to behavior.

## IMPLICATIONS FOR TREATMENT AND RESEARCH

The hypotheses that risk of endometriosis is mediated by low prenatal testosterone, and that it represents a disorder opposite to PCOS [3], have direct implications for the diagnosis, epidemiology, prevention and treatment of both disorders. The first and most important implication of the diametric hypothesis for endometriosis and PCOS is that risk factors for one disorder provide insights into potential treatments for the other disorder. This pattern can be observed in some of the current treatments for each disorder; for example, PCOS is caused in large part by excessive levels of androgens, and the symptoms of endometriosis can be alleviated by treatment with androgenic compounds, including, for example, danazol and letrozole (e.g., [67, 139]). In turn, PCOS symptoms can be reduced by treatment with flutamide, a ‘pure antiandrogen’ that antagonizes the androgen receptor [140], and anti-androgenic, estrogenic chemicals can increase endometriosis risk, as described above. Risk for PCOS is also high in women with congenital adrenal hyperplasia, who exhibit high levels of prenatal testosterone [141]; by contrast, adrenal insufficiency, which involves low testosterone, has been associated with reduced functional ovarian reserve [142], a condition that overlaps with endometriosis in its causes and correlates [143].

Table 3 provides a synopsis of current and proposed treatments for endometriosis and PCOS, that shows the diverse range of evidence for diametric treatments versus causes of the two disorders. More generally, the opposite nature of endometriosis and PCOS provides for reciprocal illumination of their causes and symptoms, such that any new finding for one condition immediately provides potential insights into the other. These insights extend to assisted reproduction; for example, women with low ovarian reserve, endometriosis, or both, may benefit from treatment with dehydroepiandrosterone (DHEA) or other androgens, which show evidence of preventing follicular atresia and facilitating fertility-related outcomes [158, 159].

**Table 3.** Evidence that causes of endometriosis represent actual or potential treatments for PCOS, and vice versa

**Treatment or cause of endometriosis or PCOS**	**Effect in endometriosis**	**Effect in polycystic ovary syndrome**	**Comments**	**References (see text for additional details)**
Danazol, a synthetic androgen with high affinity for the androgen receptor	Used to alleviate symptoms of endometriosis; administered orally or vaginally; has androgenic side effects (e.g., hirsutism)	High androgen levels are a primary cause of PCOS; flutamide, an antagonist of the androgen receptor, is used to treat PCOS	Ovarian and serum testosterone are lower in women with endometriosis than in controls, and higher in women with PCOS than in controls	[3, 49, 67, 140, 144]
Valproate, a histone deacetylase inhibitor that suppresses aromatase, used mainly to treat epilepsy and bipolar disorder	Treatment reduces endometrial lesion size in rat model and reduces proliferation of endometrial stromal cells *in vitro.* Also reduces menstrual pain in women with adenomyosis, a condition similar to endometriosis	Treatment induces major symptoms of PCOS, including increased serum testosterone	A histone deacetylase inhibitor similar to valproate, trichostatin A, induces apoptosis of endometrial cells derived from women with endometriomas	[145–147]
Oxytocin or oxytocin antagonist (i.e., Atosiban)	Atosiban treatment reduces size of endometriotic implants in rat model of endometriosis; also increases pregnancy rates in women with endometriosis undergoing embryo transfer	Oxytocin treatment alleviates obesity-related metabolic traits in rat model of PCOS, and may reduce obesity in women	Serum oxytocin levels are lower in women with PCOS and higher in women with endometriosis; oxytocin mediates food intake, metabolism and uterine smooth muscle peristalsis	[148, 149]
Letrozole, an aromatase inhibitor	Used to treat endometriosis; reduces size of endometriosis implants and pain	Used to generate PCOS in rodent models	Aromatase expression is typically elevated in endometriotic tissue, and reduced in ovarian tissue in PCOS	[150–152]
Mifepristone, a progesterone receptor antagonist	Used to treat endometriosis; reduces size of endometriosis implants and levels of pain	Used to generate PCOS in rodent models	Mifepristone increases the LH/FSH ratio and testosterone/estrogen ratio in rat model and upregulates androgen receptor in human endometrial biopsy tissue	[153]
Opiates and opiate receptor antagonists (naloxone and naltrexone)	Opioids used for pain in endometriosis also modulate HPO axis and may affect endometrial proliferation	Naloxone and naltrexone alleviate PCOS symptoms in humans and in animal models; naltrexone reduces testosterone and LH	Levels of β-endorphins are higher in PCOS, lower in endometriosis, compared to controls; opioid receptors are expressed in endometrial tissue	[154–157]

The table does not include medications for endometriosis (e.g., GnRH agonists, and oral contraceptives) that involve dampening or deactivation of the HPO axis and cessation of menstrual activity, because these do not treat the disorder itself.

Second, the hypothesis of endometriosis and PCOS as diametric disorders provides a basis for prediction and management of both conditions. Endometriosis in particular has been challenging to diagnose; the framework described here provides a large set of established and proposed clinical correlates, including, for example, low AGD, high 2D4D digit ratio, low AMH, high oxytocin, low serum testosterone, a high ratio of FSH to LH, high SHBG, low β-endorphin, low serum testosterone in the mother during early gestation, and other differences [3]. Collectively, these factors can serve as predictors of endometriosis without surgery, and, possibly, as indicators of onset, severity, form or prognosis. Early diagnosis of endometriosis is especially important because it provides the opportunity to halt progression of the disease, using GnRH agonist or combined oral contraceptive (COC) treatments that suppress natural menstrual cycling, until a reproductive opportunity, if and when desired, can be pursued. Diagnosis can also be facilitated by recognizing the expectation of transgenerational effects on endometriosis risk due to low prenatal testosterone, which would mirror the transgenerational effects, due to high testosterone, recently documented in PCOS [160].

The diametric model for endometriosis and PCOS, which follows from their evolutionary underpinnings, is in its infancy, and none of the relevant data have been collected with its predictions in mind. Box 3 provides a list of testable predictions that follow from the hypothesis. We hope that these ideas will spur further research into its scope and application, with benefits to female health, fertility and well-being, as well as leading to enhanced understanding of the diverse evolutionary bases for risks and forms of human disease.

**Conflict of interest:** None declared.

**Box 1.** Glossary of terms

Antimüllerian hormone—a glycoprotein that inhibits female reproductive structures during early development (Müllerian ducts) and regulates recruitment of follicles from the resting pool during menstrual cycling.

Anogenital distance—distance from anus to distal end of genitalia; longer in males than in females, and longer within each sex in association with higher prenatal testosterone.

Decidualization—differentiation of endometrial tissues under the influence of progesterone, in preparation for embryo implantation and trophoblast (placental) invasion, or shedding during menstruation if implantation does not occur.

Digit ratio—ratio of the 2nd to 4th finger lengths; shorter in males than females, and shorter within both sexes in association with higher prenatal testosterone.

Endometrium—highly vascularized inner lining of the uterus, that proliferates under the influence of estradiol and progesterone during the menstrual cycle.

β-endorphin—endogenous opioid peptide hormone produced in the central nervous system, that mediate pain perception, as well as GnRH secretion and other aspects of female reproduction.

Follicle stimulating hormone (FSH)—glycoprotein produced by the pituitary under the influence of GnRH that regulates development and function of the reproductive system; during the menstrual cycle, it controls growth and recruitment of ovarian follicles.

Gonadotropin releasing hormone (GnRH)—a peptide hormone released by the hypothalamus that controls production of FSH and LH in the pituitary.

Hypothalamic–pituitary–ovarian (HPO) axis—neural and hormonal system coordinating reproduction and other functions, especially via GnRH release, which affects FSH and LH, impacting ovarian cycles and menstruation.

Luteinizing hormone (LH)—glycoprotein produced by the pituitary under the influence of GnRH that triggers ovulation and the development of the corpus luteum (a temporary endocrine structure that develops from the follicle).

Menarche—a woman's first ovarian cycle resulting in menstrual bleeding or menstruation.

Menstruation—release of degraded endometrial tissue (menses) following menstrual cycle in some mammals.

Ovarian follicle—mass of cells in the ovary that contains the oocyte, as well as granulosa cells and thecal cells.

Retrograde flow—the flow of menstrual blood and endometrial cells back through the fallopian tubes, and into the peritoneal area and pelvic cavity, instead of out of the body.

**Box 2**. The primary symptoms and diagnostic features of endometriosis and PCOS

Endometriosis

Endometriosis is defined by the presence of endometrial tissue (glands and stroma) outside of the uterine cavity, at so-called ectopic (nonendometrial, noneutopic) sites. Proliferation, inflammation, and degradation of eutopic and ectopic endometrial tissue can cause dysmenorrhea (painful menstrual periods caused by strong uterine contractions, typically referred to as cramps), menorrhagia (heavy menstrual bleeding), dyspareunia (pain during intercourse), chronic pelvic pain, and reduced fertility. Symptoms can vary from minor to highly severe and debilitating, and definitive diagnosis usually requires laparoscopic examination. See Bulun et al. [5] for a useful recent review.

Polycystic ovary syndrome (PCOS)

PCOS is characterized by three primary phenotypes, (1) hyperandrogenism (high levels of testosterone in females, leading to acne, increased body or facial hair, and a lower-pitched voice); (2) polycystic ovaries, which represent ovaries that contain multiple small follicles that have stopped developing at an early stage and resemble cysts; (3) anovulation or oligo-ovulation (absence or rarity of ovulation during menstrual cycling). PCOS is highly variable among women in the presence and degree of expression of these phenotypes, and there is no consensus among medical practitioners concerning its formal definition and diagnostic criteria. This disorder is commonly, but not always, associated with obesity and insulin resistance, and many women exhibit one or more of the primary phenotypes of PCOS but not all of them. PCOS is not a discrete condition biologically, but represents a diagnostic threshold of a continuously varying set of phenotypes, that extend from ‘normality’, to variable development of components of the diagnostic criteria [6, 7]. For example, women with relatively high serum testosterone, but without PCOS, show high LH relative to FSH and low SHBG [6]. See Bellver et al. [8] and Charifson and Trumble [9] for further discussion.

**Box 3**. Testable predictions that follow from the hypotheses that: (1) low prenatal testosterone mediates the development of endometriosis and (2) endometriosis and PCOS represent diametric (opposite) disorders of the female HPO axis. The listed predictions were chosen to be amenable to robust testing, and to provide insights into both the medical and evolutionary aspects of these two conditions.

1. Women with endometriosis should exhibit higher 2D4D ratios, which are indicative of lower prenatal testosterone, compared to control women. Higher 2D4D ratio should be positively correlated with lower anogenital distance, and with lower serum and ovarian testosterone. These effects should especially apply to more-severe forms of endometriosis.
2. Females of nonhuman species, especially those that show natural menstruation (e.g., some primates, and spiny mice), should, when exposed to anti-androgenic or pro-estrogenic chemicals early in gestation, exhibit a higher incidence of endometriosis and its associated traits and correlates, as well as showing shorter anogenital distances. Similar considerations should apply to nonmenstruating animal models (e.g., mice and rats), with regard to correlates of endometriosis and proliferation of experimental ectopic implants.
3. Genetic risk of endometriosis, as calculated from GWAS summary statistics, should be positively associated with correlates and indicators of lower prenatal testosterone, including for example higher 2D4D digit ratios, lower waist to hip ratio, and lower anogenital distances. Endometriosis risk should show a negative genetic correlation with risk of PCOS.
4. Endometriosis and PCOS should be associated with sexually dimorphic facial features, as quantified morphometrically. Similar considerations should apply to additional sexually dimorphic traits, such a voice pitch and frequency, whose development is mediated in part by prenatal and postnatal testosterone.
5. Endometriosis, or high genetic or familial risk of endometriosis, should be characterized by high insulin sensitivity and other phenotypes indicative of low rates of metabolic syndrome and cardiovascular disease, compared to matched controls. This hypothesis is based on contrasts with PCOS, which typically involves high rates of obesity, type 2 diabetes, and related metabolic phenotypes [161], in association with high BMI and WHR.
6. Rates of endometriosis and PCOS appear to differ substantially among human groups from different geographic areas [162]. Human geographically based, among-group variation in 2D4D digit ratios, and anogenital distances, should parallel among-group variation in the prevalence of endometriosis and PCOS. In particular, across human groups, indicators of lower prenatal testosterone (high 2D4D, and low AGD) should be associated with higher prevalence of endometriosis and lower prevalence of PCOS, and vice versa. This variation should also be reflected in measures of serum or amniotic-fluid testosterone, during gestation.
